# ROBOTIC PANCREATODUODENECTOMY IN BRAZIL: LESSONS AFTER 15 YEARS OF THE FIRST CASE

**DOI:** 10.1590/0102-6720202400029e1822

**Published:** 2024-09-02

**Authors:** Marcos BELOTTO, Orlando Jorge Martins TORRES

**Affiliations:** 1 Hospital Israelita Albert Einstein, Department of Gastrointestinal Surgery – São Paulo (SP), Brazil;; 2 Universidade Federal do Maranhão, Hospital Universitário Presidente Dutra, Hepatopancreatobiliary Surgery and Liver Transplant, Department of Gastrointestinal Surgery – São Luís (MA), Brazil.

Pancreatoduodenectomy is a technically-challenging surgical procedure. In experienced centers, the postoperative mortality is around 5% and postoperative complications remain high, ranging from 30 to 61%^1,18^. According to Torres et al., in 52 Brazilian centers, most of hepatopancreatobiliary surgeons (65.4%) performed only open conventional pancreatoduodenectomy in 2017^15^. Robotic surgery has revolutionized minimally invasive surgical techniques, offering distinct advantages in various complex procedures, including pancreatoduodenectomy (Whipple procedure)^1,18^. It represents a significant advancement in the surgical management of various malignant and benign conditions affecting the head of the pancreas, duodenum, bile duct, and surrounding areas, especially for pancreatic head and periampullary cancer^3^. This complex procedure involves resecting the pancreas head, duodenum, bile duct, and part of the stomach, followed by the gastrointestinal tract reconstruction^3,4,14^. In pancreaticoduodenectomy, surgeon volume significantly affects outcomes, thus affecting mortality and morbidity rates, lengths of stay, and costs^2^. Tseng et al. showed that after 60 cases, the surgeon gained experience and improvement regarding blood loss, operative time, length of stay, and the achievement of negative margin resection^16^.

The robotic system provides surgeons with wristed instruments that mimic the movements of the human hand, but with greater precision and range of motion. This is particularly beneficial in the confined anatomical spaces of the pancreas and surrounding structures^6^. It also offers a three-dimensional, high-definition view of the operative field, allowing for better identification of critical anatomical landmarks and vascular structures^1,6,17^. This enhanced visualization facilitates meticulous dissection and reduces the risk of intraoperative complications^1,18^.

Robotic pancreatoduodenectomy (RPD) is safe and feasible, and in specialized centers, the procedure is associated with longer operative times and reduced intraoperative blood loss. In addition, perioperative pain scores are significantly lower with shorter lengths of stay with the robotic approach. Regarding postoperative complications, postoperative pancreatic fistula rates are similar for minimally invasive and open pancreaticoduodenectomy (OPD)^13,16^. A recent systematic review and meta-analyses by Lancellotti et al., including five studies with 12.984 patients, found that minimally invasive pancreatoduodenectomy is associated with a higher incidence of postoperative venous thromboembolism when compared to the open approach (total venous thromboembolism p<0.001; pulmonary embolism p=0.002; deep venous thrombosis p=0.004)^7^.

To date, oncological outcomes and survival are comparable between RPD and OPD. According to the current literature, RPD is either equivalent, superior, or inferior in certain aspects to OPD^16^. In approximately 15% of patients with pancreatic ductal adenocarcinoma, vascular resection (portal-mesenteric vein) is necessary^3^. Due to its complexity, occasional surgeons in low-volume centers without expertise in pancreatic surgery should not perform RPD.

The first OPD performed in Brazil was reported by Frederico Trigo Lopes in 1945 and is considered an important landmark in the country^8^. It was only in 2009 that the first RPD was performed at Hospital Israelita Albert Einstein in São Paulo^9^. Over the past 15 years, Brazil has seen a substantial increase in the adoption of robotic surgery, and it is estimated that approximately 140 thousand robotic procedures were performed across various surgical areas, including robotic pancreatoduodenectomies, reflecting the growing utilization of this advanced technique in managing pancreatic and periampullary diseases^6,12^. As of 2022, only 25.2% of the Brazilian population had private health insurance coverage, indicating that most robotic pancreatoduodenectomies occur in the private sector, where leading hospitals and medical institutions have embraced robotic surgery, supporting its integration into surgical practice^5^.

Developing cost-effective models and exploring public-private partnerships can help mitigate the financial barriers to adopting this technique^16^. However, even though the high cost of robotic systems and associated instruments (including the initial investment, maintenance, and the cost of disposable instruments used during each procedure) remains a significant barrier to widespread adoption, studies have shown that minimally invasive major pancreatic surgery entails higher intraoperative but similar overall index hospitalization costs, mainly due to reduced length of hospital stay^2^. This gap in research limits our understanding of the economic implications and potential benefits of robotic-assisted techniques for more complex pancreatic surgeries, highlighting the need for comprehensive studies to evaluate their cost-effectiveness and broader adoption^11,18^.

In the largest Brazilian series of 105 robotic pancreatic resections conducted in São Paulo from March 2018 to December 2019, 51 were pancreatoduodenectomies. Morbidity was reported in 23.8% of patients, with only one mortality. Additionally, three patients (2.8%) required conversion to open surgery. Among all patients, 24 developed pancreatic fistulas, which were treated conservatively with the late removal of the pancreatic drain. However, these data come from surgeons with expertise in pancreatic and minimally invasive surgery^10^.

And last, but not least, the complexity of robotic procedure requires extensive training and experience^6,18^. Surgeons must undergo rigorous training to achieve proficiency in robotic pancreatoduodenectomy, which can be time-consuming and resource-intensive. The steep learning curve can initially result in longer operative time and potentially higher rates of complications, including mortality, as surgeons gain experience. Establishing comprehensive training programs and centers of excellence has been crucial in building a skilled workforce capable of performing robotic pancreatoduodenectomy. Ongoing education and hands-on experience are vital for maintaining and enhancing surgical skills^1,17,18^.

The experience of over 15 years with robotic pancreatoduodenectomy in Brazil has demonstrated the significant potential of this advanced surgical technique. While challenges related to cost, accessibility, and the learning curve remain, the benefits of enhanced precision, reduced complications, and improved recovery times make robotic pancreatoduodenectomy a promising option for the management of pancreatic and periampullary diseases. Continued investment in training, research, and technological innovation will be essential for realizing the full potential of robotic surgery and expanding its impact on patient care in Brazil^7,8,10^.
